# Issues in the Preanalytical Process of Specimens for Laboratory Tests in Home Healthcare Settings

**DOI:** 10.3390/healthcare14121749

**Published:** 2026-06-17

**Authors:** Nayuta Shimizu, Kazuhiko Kotani

**Affiliations:** Center for Community Medicine, Jichi Medical University, Shimotsuke City 329-0498, Tochigi, Japan; nayuta_shimizu@jichi.ac.jp

**Keywords:** analytical process, blood test, elderly, home care, point-of-care testing

## Abstract

**Highlights:**

**What are the main findings?**

- In-home healthcare systems are necessary with the increase in vulnerable people, such as elderly patients, and laboratory tests are necessary for patient care. However, several issues in the preanalytical process, including specimen collection, specimen preservation, hemolytic phenomenon, and point-of-care testing (POCT)-related factors, can lead to inaccurate laboratory test results.
- These issues can alter the values of various analytes and hematological parameters, such as blood glucose, potassium, lactate dehydrogenase, aspartate aminotransferase, Hemoglobin A1c, hematocrit, and red cell mean corpuscular volume.

**What are the implications of the main findings?**

- Awareness of the preanalytical process among professionals involved in home healthcare is important for securing the quality of laboratory testing in these settings.
- Appropriate education and management of specimen handling and POCT among healthcare providers may help improve test quality in home healthcare settings.

**Abstract:**

Home healthcare has recently been promoted in response to the increase in vulnerable people, such as elderly patients who can have difficulty accessing clinics and hospitals in Japan. A characteristic specific to home healthcare is that laboratory tests using specimens are conducted by transport from home to laboratory centers or by point-of-care testing at home. In this case, several issues can lead to inaccurate test values. This narrative literature review summarizes issues in the preanalytical process, a critical phase for ensuring the accuracy of laboratory tests. Specimen collection may not always be smooth in the pathological conditions of some elderly patients and/or in the non-clinic/hospital environments. The preservation of specimens, considering prolonged pre-centrifugation time and storage temperature, can alter the values of various analytes, including blood glucose, potassium, and lactate dehydrogenase. In addition, hemolytic phenomenon caused by insufficient specimen collection, vibration during specimen transport, and excessive milking during fingertip blood sampling can also be an issue. Awareness of the preanalytical process in testing specimens is important for obtaining accurate laboratory tests in home healthcare settings. This comprehensively summarized paper will be helpful in securing test quality and patient care.

## 1. Introduction

The number of vulnerable people, such as elderly patients, is increasing in developed countries, including Japan, which is classified as a super-aged society [1]. The situation of society is driving social changes. In response to the needs of such patients wishing to receive healthcare at home, who have difficulty accessing clinics and hospitals, home healthcare has been increasingly required in recent years [1].

**Figure 1:**
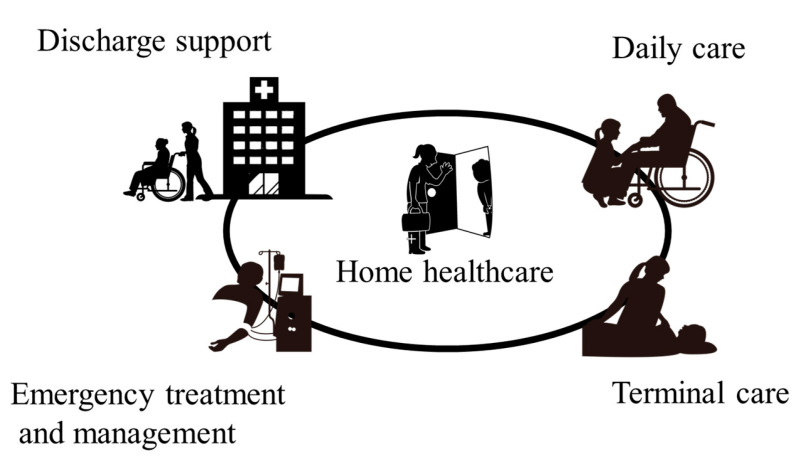
Fundamental components of home healthcare.

Home healthcare consists of some fundamental components in the community-based systems (Figure 1). The components include discharge support to facilitate the transition from the hospital to the home, ongoing daily care, regular home visits by physicians and nurses, emergency treatment and management to sudden changes and exacerbation of a patient’s condition during home healthcare, and terminal care that respects the patient’s dignity.

In typical settings of home healthcare, patients live with cancer, chronic heart failure, chronic respiratory disease, chronic kidney disease, cardiovascular disease, cerebrovascular disease, and dementia at the end of their life [1]. In such patients, while extensive laboratory tests might not be necessary [2], regular monitoring with routine laboratory tests [1,2] or diagnostic tests for hospital admission due to acute exacerbation of chronic diseases [1] is desired. Furthermore, routine laboratory tests using blood have been reported as clinical indicators in these patients. For example, low levels of albumin [3,4], hemoglobin [3,5], and estimated glomerular filtration rate [6], as well as high levels of aspartate aminotransferase (AST) [3,7] and C-reactive protein [8], are reported to be prognostic indicators in elderly patients receiving home healthcare. Thus, laboratory tests using specimens including blood, urine, and other biological samples have been conducted in home healthcare settings [1].

A characteristic specific to home healthcare is that laboratory tests using specimens transported from the patient’s home to laboratory centers (in medical institutions or external centers) for their analysis or are analyzed in the patient’s home by point-of-care testing (POCT) [2]. In laboratory tests in home healthcare settings, there are several issues that are known to occur during the preanalytical process. Of note, most errors in laboratory tests generally occur during the preanalytical process rather than during the analytical process [9,10]. Inadequate preanalytical process may result in inaccurate test results. In fact, recent international guidance for laboratory centers is based on ISO 15189 [11] accreditation, in which the preanalytical process must be checked to ensure test quality [5]. Ideally, specimens should be smoothly collected and promptly measured after collection and centrifugation [2,10]. Specimen collection may not always be smooth in the pathological conditions of some elderly patients and/or in the non-clinic/hospital environments. The preservation of specimens during transport involves prolonged storage and produces storage-temperature-related changes in analytes [2]. Hemolytic phenomenon during blood collection and transport is problematic in laboratory tests in home healthcare settings. Moreover, although POCT can avoid the need for specimen transport, its operation and specimen handling still require attention [12].

Importantly, preanalytical errors by humans can be prevented [10]. It is essential for all professionals involved in home healthcare to be aware of the preanalytical process. This paper summarizes the issues in the preanalytical process; then, this comprehensively summarized information would be helpful for improving the accuracy of tests conducted in home healthcare settings.

## 2. The Issues in Preanalytical Process in Home Healthcare Settings

**Figure 2:**
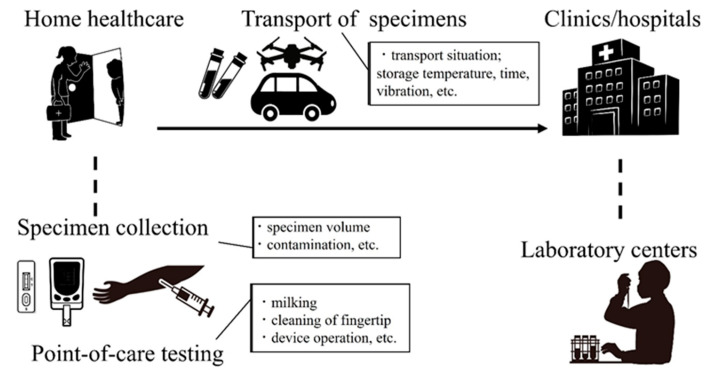
Points related to issues associated with inaccurate testing during the preanalytical process in home healthcare settings.

In our knowledge and experience, the issues regarding the preanalytical process in home healthcare settings are shown in Figure 2. In general, depending on traffic, it is necessary to transport specimens from patients’ homes to laboratory centers [2]. The time required for measurement, storage temperature, and vibration should be considered when transporting specimens [2,9]. The following are considered to be the key thematic points of the issues: (1) specimen collection, (2) preservation of specimens, (3) hemolytic phenomenon, and (4) POCT-related factors.

## 3. Specifics to the Issues

For specifics on the issues, we collected information on these points via a targeted literature search. PubMed and Web of Science were searched for articles published from 1 January 2000 to 31 May 2026. Studies not published in English, letters, and conference papers were excluded from the search.

For (1) specimen collection factors, the search included a combination of terms related to specimen collection, insufficient specimen volume, collection tubes, anticoagulants, and equipment contamination. The PubMed search terms were as follows: (“Blood Specimen Collection” [Mesh]) AND (“insufficient specimen volume” OR “collection tubes” OR insufficient OR underfilled OR “Anticoagulants” [Mesh] OR “Equipment Contamination” [Mesh]). The Web of Science search terms were as follows: TS = (“blood collection” OR “blood sampling” OR phlebotomy) AND (“insufficient specimen volume” OR “underfilled tube” OR “underfilled tubes” OR anticoagulant* OR “equipment contamination” OR “sample contamination” OR “specimen contamination”). The search yielded 231 candidate records from PubMed and 471 from Web of Science. After reviewing the identified records, we excluded duplicate records (n = 48), records without abstracts (n = 7), and records not involving human subjects (n = 75). We also excluded records that were not related to errors in blood collection (n = 393) and those not relevant to blood collection in home healthcare settings (n = 168). Finally, 11 articles were included in this review [13,14,15,16,17,18,19,20,21,22,23]. Among the eleven articles, eight were original articles [13,14,15,16,17,18,19,20], two were reviews [21,22], and one was an opinion paper [23].

For (2) specimen preservation factors, the search included a combination of terms related to the time or temperature of specimen preservation. The PubMed search terms were as follows: (“Blood Preservation” [Mesh] OR “Preservation, Biological” [Mesh]) AND (“Blood Chemical Analysis” [Mesh] OR “Clinical Chemistry Tests” [Mesh]) AND (time OR temperature). The Web of Science search terms were as follows: TS = (“blood preservation” OR “biological preservation” OR “sample preservation” OR “specimen preservation” OR “biospecimen preservation”) AND (time OR temperature). The search yielded 119 candidate records from PubMed and 372 from Web of Science. After reviewing the identified records, we excluded duplicate records (n = 2), records without abstracts (n = 5), and records not involving human subjects (n = 310). We also excluded studies that were not related to errors in specimen preservation (n = 111) and studies not relevant to home healthcare settings (n = 58). Finally, five studies were selected for inclusion in this review [24,25,26,27,28]. All five studies were original research articles.

For (3) hemolytic phenomenon factors, the search included a combination of terms related to hemolysis and routine laboratory tests. The PubMed search terms were as follows: “(Blood Chemical Analysis” [MeSH]) AND (routine OR common) AND (“Hemolysis” [MeSH]). The Web of Science search terms were as follows: TS = (“blood chemical analysis” OR “blood chemistry” OR “clinical chemistry” OR “laboratory test*”) AND (routine OR common) AND (hemolysis OR hemolysis). The search yielded 51 candidate papers from PubMed and 165 from Web of Science. After reviewing the identified records, we excluded duplicate records (n = 11), records without abstracts (n = 1), and records not involving human subjects (n = 9). We also excluded studies that were not related to the effects of specimen hemolysis on routine laboratory test results (n = 153) and studies not relevant to home healthcare settings (n = 38). Finally, four studies were selected for inclusion in this review [29,30,31,32]. Among the four studies, three were original articles [29,30,31], and one was a review article [32].

For (4) POCT-related factors, the search included a combination of POCT and preanalytical issues. The PubMed search terms were as follows: “Point-of-Care Testing” [Mesh] AND (“Blood Specimen Collection” [Mesh] OR “Pre-Analytical Phase” [Mesh]). The Web of Science search terms were as follows: TS = (“point-of-care testing” OR POCT OR “point of care testing”) AND (“blood specimen collection” OR “blood sample collection” OR “blood collection” OR “pre analytical” OR “pre-analytical”). The search yielded 32 candidate papers from PubMed and 75 from Web of Science. After reviewing the identified records, we excluded duplicate records (n = 4), records without abstracts (n = 3), and records not involving human subjects (n = 5). We also excluded studies that were not related to pre-analytical errors in POCT (n = 37) and studies not relevant to home healthcare settings (n = 56). Finally, two studies were selected for inclusion in this review [33,34]. Among these two studies, no original articles were identified; one study was a review article [33], and the other was an opinion paper [34].

### 3.1. Specimen Collection

Among the 11 articles, the most frequent topics were insufficient or incomplete filling of blood collection tubes [13,17,18,19,20] and specimen contamination by blood collection tube-derived substances, such as EDTA or K2EDTA [14,15,16,22,23]. The other topics included hemolysis risks [21].

In Japan, patients receiving home healthcare are often elderly, and it is difficult to sample blood with a sufficient specimen volume in some elderly patients with pathological conditions (i.e., dehydration, edema due to chronic heart failure, and renal failure) [13]. The effects of specimen collection errors were reported. Insufficient blood filling in K_2_-EDTA tubes can lead to increased potassium [14,15,16] and glycated hemoglobin (HbA1c) levels [17], as well as decreased hematocrit and mean corpuscular volume (MCV) of red cells [18]. Inadequate blood filling in heparin tubes may result in elevated levels of lactate dehydrogenase (LD), potassium, and AST [13]. Insufficient blood filling causes hemolysis [19,20,21]. Hemolysis may also be induced by excessive negative pressure during collection [19,21], use of a thin needle [22], or contamination with disinfectant alcohol [22]. The improper order of blood draw may cause additive contamination from the blood collection tubes previously used, which leads to erroneous test results [10]. Backflow contamination after the use of K-EDTA tubes can result in increased potassium [14] and decreased LD levels [16]. In home healthcare settings, unlike hospital or outpatient laboratory settings, prompt assessment of specimen quality and recollection are often difficult. Therefore, in some cases, blood collection should not be performed. When venous access is challenging, most tests should be considered to be influenced.

### 3.2. Preservation of Specimens

Prolonged storage of blood can lead to changes in analytes. The effects of time and temperature on whole-blood preservation have been previously reported. Blood glucose levels decrease because of increased glycolysis if sodium fluoride tubes are not used [24]. Reduced Na+/K+-ATPase activity results in increased potassium levels [25,26]. Altered cell membrane activity causes elevated LD levels [25,26,28]. Changes are seen in the platelet count [27], white blood cell count [27], and MCV [24]. When specimens need to be transported from patients’ homes to laboratory centers in home healthcare settings, temperature control according to the specimen type and target analytes is an important consideration [2].

### 3.3. Hemolytic Phenomenon

As mentioned above, blood collection is a possible cause of the hemolytic phenomenon. In addition, vibration during specimen transport increases the incidence of hemolysis [9]. The effect of hemolysis on clinical chemistry analytes in home healthcare settings has been reported. Lactate dehydrogenase [29,30,31], potassium [29,30,31], and AST [29,30,31] showed elevated levels, even under mild hemolysis. Hemolysis causes the release of intracellular components from red blood cells, resulting in elevated levels of these analytes [32,35]. In home healthcare settings, local road and traffic conditions should be considered during specimen transport. To suppress excessive vibration, it is recommended that collection tubes be transported with their bottoms inserted into polystyrene holders rather than using wire or plastic collection tube holders, which are more prone to vibration. As part of practical quality management for specimens in home healthcare settings, laboratories should define acceptance and rejection criteria for specimens, including hemolysis, and communicate these criteria to healthcare staff involved in home specimen collection. In accordance with ISO 15189:2022 [11], these criteria should be documented as part of the sample receipt procedure. For hemolysis-related quality indicators, the IFCC Model of Quality Indicators, such as the proportion of samples with free hemoglobin >0.5 g/L, may be used as a reference [36], and quality targets can be established based on the analyte, local validation, and baseline performance.

### 3.4. POCT-Related Factors

As well as having the advantage of providing immediate measurements in the patient’s home, POCT can reduce transport-related preanalytical risks [2]. However, there are preanalytical issues associated with POCT [12,37]. The effects of preanalytical issues with POCT have been reported. When a lancet is used to collect a small amount of blood from the fingertip, excessive milking can cause hemolysis or dilution of the specimen with interstitial fluid [33]. In POCT systems that process whole-blood specimens in closed cartridges, hemolysis cannot be visually detected; therefore, particular caution is required, especially when the POCT device is not equipped with a hemolysis detection system [34]. Inadequate cleaning of the fingertip may result in inaccurate blood glucose levels due to contamination of food with glucose and ascorbic acid [33]. In immunochromatography, insufficient specimen volume can lead to erroneous results [33]. As immunochromatographic test kits may not function adequately at low temperatures, the kits stored in a refrigerator should be brought to room temperature before use. In addition, as immunochromatography is usually interpreted visually, there is a risk of misinterpretation under a weak lighting condition in the patient’s home [33]. When the reaction line is faintly positive, misinterpretation as negative has also been reported more frequently among operators aged 40 years or older, likely due to age-related decline in visual sensitivity [33]. In cases where household lighting is weak, the use of a desk lamp or the introduction of devices with automated reading functions would be necessary [33]. For POCT devices that require the insertion of a reagent cassette or strip, proper placement must be verified to ensure accurate performance [33]. The package inserts for POCT devices and test kits include precautions tailored to the methods and measurements of each test. It is essential to review the procedures when using these devices and kits for the first time. Device management should be implemented, including regular inspection and calibration of POCT devices for quality assurance [37,38]. Additional POCT-related factors include the currently limited range of laboratory tests that can be measured by POCT and the relatively high cost of POCT kits.

## 4. Countermeasures of the Issues

Countermeasures are needed for these issues. Increasing awareness of the preanalytical process in laboratory tests using specimens in home healthcare settings is essential. Education on test quality in the process among home healthcare professionals must be continued. The standardization of sampling error detection and the development of standard operating procedures can also contribute to preventing preanalytical errors [39]. It would be optimal that management of the preanalytical process is widely and thoroughly implemented as described in the ISO 15189: 2022 accreditation [40]. When operators visiting patients’ homes are not laboratory professionals, it is desirable that laboratory professionals provide information on the preanalytical process through prior explanation and hands-on training [33,34]. Because POCT is commonly performed by non-laboratory professionals, particular attention should be paid to it [12]. Operator variability should be minimized through structured training and competency assessment [34].

## 5. Future Perspectives

Countermeasures for these issues might come from different future directions. The use of drones for specimen transport represents a practical alternative [41], and this may be applied in home healthcare settings. Studies have already examined whether drones are suitable for routine laboratory testing with immediate transport; in particular, during disasters, drone transport is documented to help avoid delays caused by traffic conditions and serve as a valuable means of delivering medical materials [41,42]. The use of drones has some weak points; for instance, it is prudently adopted when considering bad weather, more payload, flight distance, and airspace regulations. The security of specimens during transport is also discussed. The establishment of guidelines with scientific evidence will be of importance in its clinical use [41,42]. Further multicenter studies are needed to evaluate analyte stability under realistic home transport conditions, including transport time, temperature changes, and vibration.

Another future direction is the strategic integration of artificial intelligence (AI) tools, which may also improve the preanalytical process [37,43]. Image-based AI tools using mobile cameras may enable real-time detection of specimen labeling errors or visible sample abnormalities, even in home healthcare settings [43]. However, the lack of standardized methods for evaluating parameters in the preanalytical process makes it difficult to develop AI algorithms [43]. In addition, cybersecurity concerns and algorithm explainability remain important challenges [37]. Studies evaluating workflow efficiency and cost-effectiveness are also needed to determine whether AI tools can be sustainably implemented in routine home healthcare practice.

## 6. Limitations

Several limitations should be acknowledged. First, because this was designed as a narrative literature review, the search strategy, study selection, and synthesis may have search bias. The conclusions of this review should therefore be interpreted as a qualitative overview. Second, because only articles published in English were included, relevant studies published in other languages may have been missed. Third, the number of studies directly examining preanalytical errors in home healthcare settings was small, making it difficult to quantitatively compare the magnitude of preanalytical errors or their clinical consequences across studies. For the same reason, we could not fully suggest the training requirements for each type of operator or perform a risk ranking of preanalytical factors based on clinical impacts. To make further evidence on preanalytical errors in laboratory testing in home healthcare settings, future multicenter studies on specimen transport conditions from patients’ homes, as well as randomized or pragmatic comparative studies of POCT and central laboratory testing, might be needed.

## 7. Conclusions

Improvement of the preanalytical process for laboratory tests using specimens is important for ensuring the test quality in home healthcare settings. This, in turn, contributes to a precise diagnosis and treatment in home healthcare. The idea of laboratory tests in home healthcare continues to grow in super-aged societies, such as Japan. This comprehensively summarized information is available to ensure the test quality in this field. Further studies are needed to accumulate evidence in this field.

## Data Availability

No new data were created or analyzed in this study.

## Abbreviations

The following abbreviations are used in this manuscript:

AST	aspartate aminotransferase
POCT	point-of-care testing
MCV	mean corpuscular volume
LD	lactate dehydrogenase
